# Impact of newborn screening on outcomes and social inequalities in cystic fibrosis: a UK CF registry-based study

**DOI:** 10.1136/thoraxjnl-2019-213179

**Published:** 2019-11-26

**Authors:** Daniela K Schlüter, Kevin W Southern, Carol Dryden, Peter Diggle, David Taylor-Robinson

**Affiliations:** 1 Centre for Health Informatics, Computing and Statistics, Lancaster Medical School, Lancaster University, Lancaster, UK; 2 Department of Women’s and Children’s Health, University of Liverpool, Liverpool, UK; 3 Department of Paediatrics, Wishaw General Hospital, Wishaw, UK; 4 Department of Public Health and Policy, University of Liverpool, Liverpool, UK

**Keywords:** cystic fibrosis, bacterial infection, respiratory infection

## Abstract

**Background:**

Newborn bloodspot screening (NBS) for cystic fibrosis (CF) was introduced across the UK in 2007 but the impact on clinical outcomes and health inequalities for children with CF is unclear.

**Methods:**

We undertook longitudinal analyses of UK CF registry data on over 3000 children with CF born between 2000 and 2015. Clinical outcomes were the trajectories of percent predicted forced expiratory volume in one second (%FEV_1_) from age 5, weight for age and body mass index (BMI) SD-scores from age one, and time to chronic *Pseudomonas aeruginosa* (cPA) infection. Using mixed effects and time-to-event models we assessed the association of NBS with outcomes and potential interactions with childhood socioeconomic conditions, while adjusting for confounders.

**Results:**

NBS was associated with higher average lung function trajectory (+1.56 FEV_1_ percentage points 95% CI 0.1 to 3.02, n=2216), delayed onset of cPA, and higher average weight trajectory intercept at age one (+0.16 SD; 95% CI 0.07 to 0.26, n=3267) but negative rate of weight change thereafter (−0.02 SD per year; 95% CI −0.03 to −0.00). We found no significant association of NBS with BMI or rate of change of lung function. There was no clear evidence of an impact of NBS on health inequalities early in life.

**Conclusions:**

Children diagnosed with CF by NBS in the UK have better lung function and increased early weight but NBS does not appear to have narrowed early health inequalities.

**Key messages**

**What is the key question?**

- How has newborn screening for cystic fibrosis (CF) affected early outcomes; and has screening reduced the well documented social inequalities in CF outcomes, whereby children growing up in more disadvantaged circumstances experience worse outcomes?

**What is the bottom line?**

- Newborn screening for CF is associated with better early weight and lung function outcomes as well as delayed onset of chronic *Pseudomonas aeruginosa* (cPA) infection but screening does not seem to have reduced inequalities.

**Why read on?**

- Using UK CF registry data from over 3000 children born in the new millennium with up to 15 years of follow-up, we show that lung function, cPA infection and early weight measures are better in the screened compared with the clinically diagnosed population. However, inequalities persist whereby children from the most deprived areas still have worse nutritional and lung function status.

## Introduction

Cystic fibrosis (CF) is a serious inherited condition. One in 2500 babies born in the UK have CF, with over 10 000 people living with CF in the UK today. CF is caused by variants of the CF transmembrane conductance regulator (*CFTR*) gene, resulting in the build-up of mucus in various organs, in particular those of the respiratory and digestive systems. Most people with CF die prematurely from their disease through respiratory failure. In the 1960s median survival in the UK was estimated to be below 10 years of age.^1^ Over the past decades, outcomes have improved due to multidisciplinary care, nutritional support and new treatments, such that the median survival for a child with CF born in the UK today is estimated to be 47 years.^2^

Most children in the UK are diagnosed within the first few weeks of life through newborn bloodspot screening (NBS). Population-based NBS for CF first became feasible in the late 1970s when elevated levels of immunoreactive trypsin (IRT) in dried bloodspot samples from CF positive babies were detected. In the 1980s NBS for CF was first introduced in regions in England (East Anglia, Leeds, Northampton, the West Midlands and the Trent region) alongside screening in Northern Ireland; in the 1990s it was extended to Wales.^3^ At the start of February 2003 screening was introduced in Scotland, followed by the universal roll out in England in April 2007. The areas covered before universal screening was introduced in 2007 were large, and heterogeneous in terms of poverty and social conditions.

The number of children diagnosed through NBS has risen year on year in the UK, such that in 2017, out of 214 newly diagnosed cases of CF, 80% (172 individuals) were identified by NBS.^2^ Prior to universal rollout in 2007 a variety of screening approaches were used in the different regions, based on an initial IRT measurement from a dried bloodspot taken in the first week of life followed by DNA analysis. After 2007, the national programme has resulted in a more consistent approach that includes IRT measurement, DNA analysis and a sweat test (for more detail see 4). The programme has sensitivity of 96% and positive predicted value of 0.76.

The rationale for NBS is to facilitate early intervention, before lung disease is established, which in turn may lead to long-term benefits for people with CF; however, the evidence base to support this public health strategy is not robust.^5^ The Cochrane review of randomised controlled trials of NBS reported a transient improvement in nutritional outcomes, but clinical trials examining NBS for CF have not demonstrated clear improvements in respiratory condition and in some cases have suggested potential for harm with earlier airways infection.^6^ A review of observational registry based studies supports improved nutrition and early lung function outcomes, but less evidence for effects on survival.^7^ In this study, we use the UK CF registry data to assess the impact of early diagnosis by NBS on outcomes in CF in the UK.

Adverse socioeconomic conditions (SECs) in childhood are associated with a range of poor health outcomes in the UK and internationally.^8^ For children with CF, inequalities in outcomes are evident early in life, whereby low SECs are associated with lower birth weight, worse nutritional status, lung function and survival.^9–11^ Universal interventions such as screening have the potential to reduce health inequalities if they have a differential impact, improving health outcomes to a greater extent in more disadvantaged groups. NBS for CF in the UK ensures that all children are diagnosed within the first weeks of life. This removes any differential time to diagnosis and differential disease progression prior to diagnosis by socioeconomic status, whereby more disadvantaged children may become sicker prior to diagnosis in unscreened populations. In this study, we assessed whether NBS for CF has an impact on inequalities in early health outcomes.

## Methods

### Study design, setting, data sources and participants

We carried out longitudinal and time to event analyses of lung function, infection and nutritional status in individuals with CF captured in the UK CF registry. Individuals born between 2000 and 2015 were included but lung function measurements taken before the age of five were excluded as forced expiratory volume in one second (FEV_1_) was not routinely recorded before this age. Measures of nutritional status before the age of one were excluded due to non-linear behaviour in this period.^10^ Data from post-transplant review visits were also excluded.

The UK CF registry collects data from patient annual reviews and is estimated to capture 99% of the current UK CF population.^12^ The stepwise introduction of NBS through regional programmes and the capture of all individuals with CF in the registry, those diagnosed by NBS and those diagnosed clinically, enabled us to evaluate NBS as a natural experiment.

### Outcome, exposure and covariates

The outcomes of interest were weight-for-age SD-scores and body mass index (BMI) SD-scores from age one (see online supplementary figure S1 and S2); and lung function from age 5 as measured by percent predicted FEV_1_ (%FEV_1_) based on the Global Lung Initiative (GLI) reference equations^13^ (see online supplementary figure S3). We also assessed time to chronic *Pseudomonas aeruginosa* (cPA) infection. In the UK CF registry cPA is recorded as having three or more positive isolates in the last 12 months.

10.1136/thoraxjnl-2019-213179.supp1 Supplementary data

The primary exposure of interest was diagnosis by NBS. The UK CF registry includes a question on mode of diagnosis which captures whether patients were diagnoses by NBS. The second exposure of interest was childhood socioeconomic conditions. We used the Index of Multiple Deprivation (IMD), a small area deprivation measure, based on the first postcode recorded in the registry, as a proxy for childhood socioeconomic conditions. The IMD is a measure of relative deprivation which combines information on income, employment, health, education, access to services, crime/safety and environment/housing. The domains vary slightly between England, Wales, Scotland and Northern Ireland (for more information see 14–17). In order to obtain a comparable measure of deprivation across England, Wales, Scotland and Northern Ireland, we calculated country-specific IMD SD-scores, which we used in our analysis. More details are given in online supplementary material section S2.

We adjusted for the following covariates and potential confounders: year of birth (continuous covariate to capture cohort effects over time), age, pancreatic insufficiency (PI, coded as 0 or 1 according to whether pancreatic enzyme supplements were ever prescribed), genotype (coded as the number of F508del alleles (0, 1 or 2)), sex, ethnicity (white/other) and diagnosis by meconium ileus.

### Statistical analysis

#### Estimation of the effect of diagnosis by NBS on health outcomes

Following our previous approach^10 18^ we modelled the longitudinal trajectories of weight for age SD-scores, BMI SD-scores and %FEV_1_ using linear mixed effects models for each outcome with random intercept and random slope. We used age as the time scale with the intercept being at 1 and 5 years for weight/BMI and %FEV1, respectively. The modelled trajectories are therefore straight lines characterised by their (intercept) values at age one or five (for weight/BMI and %FEV1, respectively) and their subsequent slope. In the analysis we assessed whether the population average outcome trajectories in the group diagnosed by NBS were higher or lower than those of the clinically diagnosed group (NBS main effect). We also assessed whether the rate of change over time (slope) differed between the two groups (NBS: age interaction effect); if this is the case, the main effect gives the population average difference between the two groups at age one/five and the interaction effect indicates how this difference changes for each year of age.

We initially fitted a baseline model in which we adjusted for all covariates and potential confounders. We then fitted two further models, one that additionally included the NBS main effect only and one that included the NBS main effect and an interaction between NBS and age to assess whether NBS had an effect on the rate of change of the outcome. We then selected the best fitting model according to the results of likelihood ratio tests (LRTs) testing for significance at the conventional 5% level.

To assess whether diagnosis by NBS was associated with changes in time to acquiring cPA infection, we modelled event times using a Weibull model. We again initially fitted a baseline model and then included an NBS main effect. Significance was again assessed with a LRT. Onset of cPA was interval censored in our dataset, which can be easily accommodated in parametric survival models. Results are reported using the accelerated failure time parameterisation.

#### Estimation of inequalities in outcomes and the effect of NBS on these inequalities

To assess whether SECs were associated with outcomes in our study population we used the final models for each outcome developed above as new baseline models. We then followed the same approach as above; for the longitudinal outcomes, we fitted two further models, one with an additional deprivation main effect only and one with a deprivation main effect and an interaction between deprivation and age. For cPA we fitted one additional model with a deprivation main effect. We again selected the best fitting model based on the results from LRTs.

To assess whether the introduction of NBS had reduced any inequalities in outcomes, we then added interaction terms between deprivation and diagnosis by NBS in the final models if the models included both deprivation and NBS effects. Significant interaction terms would indicate that the effect of diagnosis by NBS differed by SECs. We also compared the association of SECs with outcomes in the clinically diagnosed compared with the NBS population.

We used R version 3.5.0 and the packages nlme^19^ and survival^20^ for the analyses. See online supplementary material section S3 for the model equations.

### Robustness tests

The CF population born post-2007 and not diagnosed by NBS is primarily composed of individuals diagnosed before the NBS result was available (either clinically through meconium ileus or from a family history), individuals that were born in another country where screening for CF was not implemented, and false negatives. To ensure that the inclusion of this diverse set of individuals as the comparison group in the recent years had not led to biassed estimates, we repeated the analyses to estimate the effect of NBS including only children born between 2000 and 2007.

Individuals diagnosed with meconium ileus were in the non-NBS group, which disproportionately placed those with worse clinical status in this group. Although we adjusted for this in the main analysis, we conducted another robustness test in which we excluded all individuals diagnosed by meconium ileus. We also repeated all analyses estimating the effect of NBS, SECs and the interaction of the two, using propensity score adjustment, on its own and together with covariate adjustment. Propensity scores were calculated using a logistic regression model for the probability of being diagnosed by NBS conditional on year of birth, meconium ileus, sex, genotype, ethnicity and PI.

Finally, we repeated our time to event analysis for cPA using different model specifications (exponential and log-logistic models) in order to check that the findings were robust to different distributional assumptions.

### Role of the funding source

The corresponding author had full access to all the data in the study and final responsibility for the decision to submit for publication.

## Results

### Population characteristics

**Figure 1:**
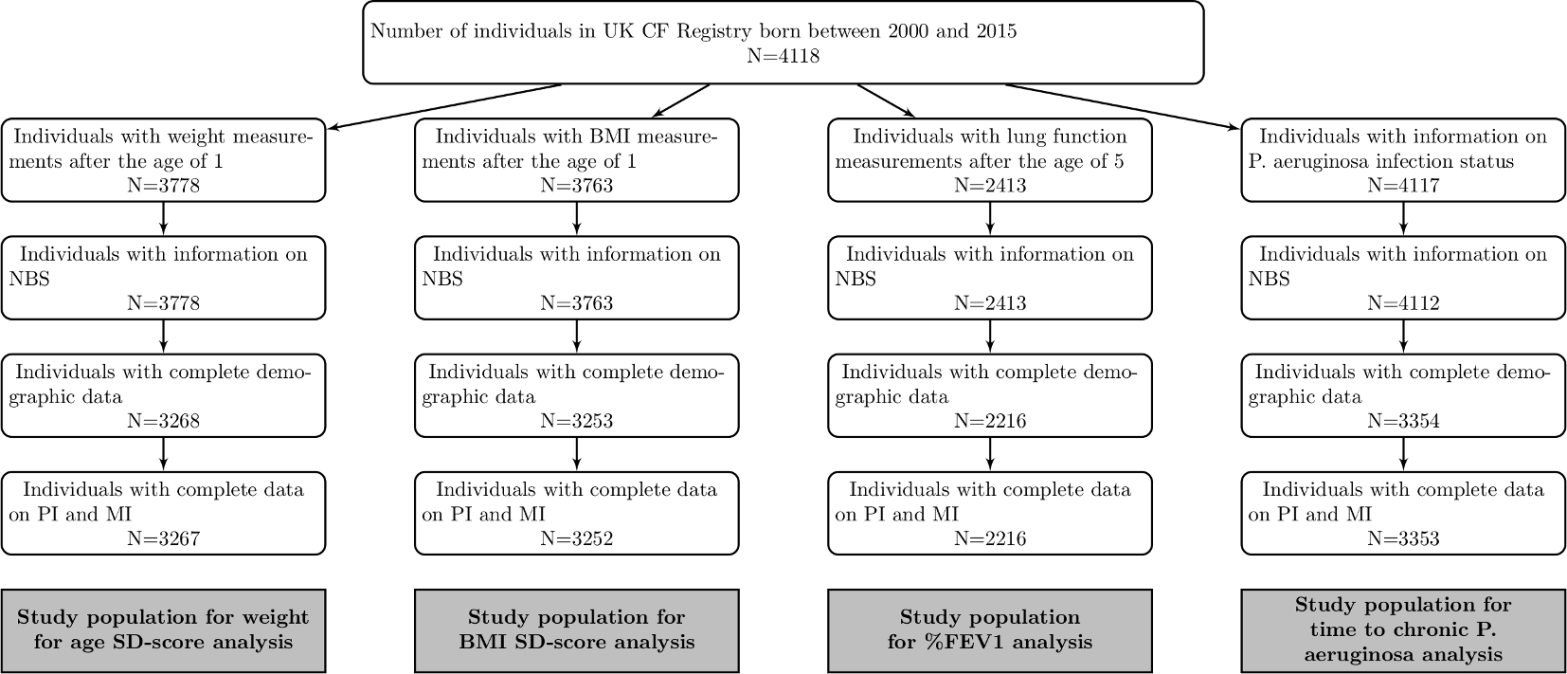
Flow diagram of the selection process for the study populations. BMI, body mass index; CF, cystic fibrosis; FEV_1_, forced expiratory volume in one second; NBS, newborn bloodspot screening; PI, pancreatic insufficiency; MI, Meconium Ileus.

The UK CF registry included 4118 children born between 2000 and 2015 (figure 1). A total of 3267 individuals with 20 606 measurements and a median of six measurements per individual were included in the analysis of weight for age SD-scores. For the analysis of BMI we included 3252 individuals with a total of 20 357 measurements and a median of six measurements per individual. In both the weight and the BMI study populations 45% of the children were diagnosed by NBS. The median age at diagnosis in the NBS groups was 0.07 years compared with 0.14 years in the groups diagnosed clinically. For %FEV_1_ we included 2216 individuals in the analysis with a total of 10 287 measurements and a median of four measurements per individual. 35% of these children were diagnosed by NBS and median age at diagnosis was 0.07 years in the NBS group compared with 0.24 year in the clinically diagnosed group. 3353 individuals were included in the analysis of time to cPA infection out of which 603 individuals developed cPA; 46% were diagnosed by NBS. Median age at diagnosis was 0.07 years and 0.13 years in the group diagnosed by NBS and diagnosed clinically, respectively.

The distribution of most covariates and potential confounders was comparable between the screened and unscreened groups (table 1). As expected, the proportion of individuals born before and after 2007 differed between groups, as did the proportion diagnosed by meconium ileus. Mean weight SD-score between the ages 1 and 2 was 0.26 (diagnosed by NBS; corresponds to 60th centile on growth chart) compared with 0.07 (diagnosed clinically; corresponds to 53rd centile on growth chart). There was a similar improvement in BMI SD scores (0.7 compared with 0.58 in the NBS and clinically diagnosed groups, respectively, which corresponds to the 76th and 72nd centile on the growth chart). Mean %FEV_1_ between the ages 5 and 6 was 89.22 in those diagnosed by NBS compared with 87.88 in individuals diagnosed clinically. See online supplementary material section S4 for tables of demographics stratified by year of birth pre-2007 and post-2007.

**Table 1:** Characteristics of the study populations for each clinical outcome stratified by diagnosis by NBS

	Weight		BMI		%FEV_1_		cPA	
	Clin. diag.	Diag. by NBS	Clin. diag.	Diag. by NBS	Clin. diag.	Diag. by NBS	Clin. diag.	Diag. by NBS
N	1799	1468	1792	1460	1444	772	1827	1526
Age range in years	1–15.9	1–15.6	1–15.8	1–15.9	5–15.9	5–15.6	0–15.9	0–15.9
Male (%)	904 (50.3)	757 (51.6)	900 (50.2)	754 (51.6)	712 (49.3)	401 (51.9)	920 (50.4)	787 (51.6)
Non-white ethnicity (%)	116 (6.4)	53 (3.6)	116 (6.5)	52 (3.6)	87 (6.0)	32 (4.1)	116 (6.3)	57 (3.7)
Born abroad (%)	102 (5.7)	35 (2.4)	101 (5.6)	35 (2.4)	81 (5.6)	27 (3.5)	102 (5.6)	39 (2.6)
F508 class (%)								
Homozygous	1005 (55.9)	756 (51.5)	1004 (56.0)	755 (51.7)	821 (56.9)	398 (51.6)	1016 (55.6)	789 (51.7)
Heterozygous	608 (33.8)	595 (40.5)	603 (33.6)	589 (40.3)	478 (33.1)	305 (39.5)	625 (34.2)	615 (40.3)
Other	186 (10.3)	117 (8.0)	185 (10.3)	116 (7.9)	145 (10.0)	69 (8.9)	186 (10.2)	122 (8.0)
IMD z-score (median (IQR))	−0.29 (−0.79- 0.53)	−0.29 (−0.79- 0.66)	−0.29 (−0.79- 0.53)	−0.29 (−0.79- 0.66)	−0.29 (−0.79- 0.53)	−0.29 (−0.79-0.53)	−0.29 (−0.79- 0.53)	−0.29 (−0.79- 0.66)
Meconium Ileus (%)	596 (33.1)	0 (0.0)	593 (33.1)	0 (0.0)	425 (29.4)	0 (0.0)	611 (33.4)	0 (0.0)
Pancreatic insufficient (%)	1592 (88.5)	1227 (83.6)	1589 (88.7)	1223 (83.8)	1293 (89.5)	659 (85.4)	1614 (88.3)	1276 (83.6)
Born pre-2007 (%)	1192 (66.3)	289(19.7)	1192 (66.5)	289 (19.8)	1174 (81.3)	287 (37.2)	1193 (65.3)	289 (18.9)
Median age at diagnosis in years (IQR)	0.14 (0–7.82)	0.07 (0–0.42)	0.14 (0–7.84)	0.07 (0–0.42)	0.24 (0–8.45)	0.07 (0–0.5)	0.13 (0–7.75)	0.07 (0–0.41)
Mean outcome (SD) between the ages 1 and 2 for weight and bmi and ages 5 and 6 for %FEV_1_	0.07 (1.05)	0.26 (1.03)	0.58 (1.11)	0.7 (1.03)	87.88 (16.38)	89.22 (15.76)	NA	NA
Mean FVC between ages 5 and 6 (SD)	NA	NA	NA	NA	93.37 (17.24)	94.66 (15.46)	NA	NA
Median FEV_1_/FVC between ages 5 and 6 (IQR)	NA	NA	NA	NA	0.96 (0.89–1.02)	0.96 (0.89–1)	NA	NA

BMI, body mass index; cPA, chronic Pseudomonas aeruginosa; FEV_1_, forced expiratory volume in one second; FVC, forced vital capacity; NBS, newborn bloodspot screening.

**Figure 2:**
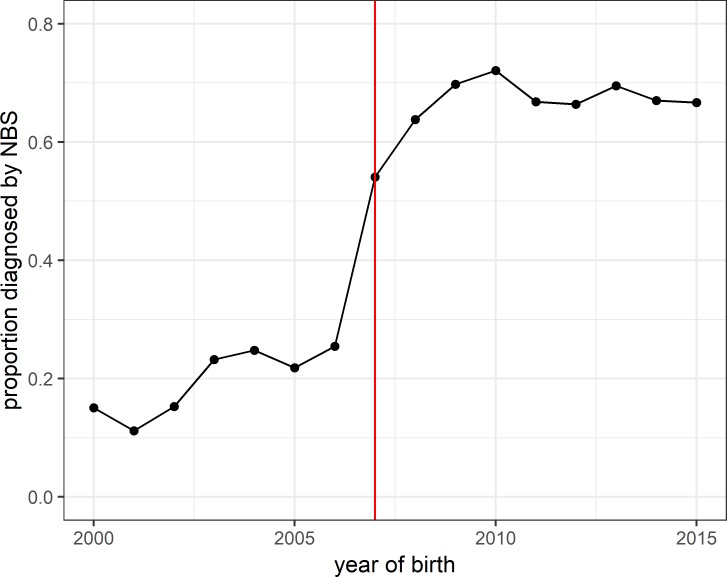
Proportion diagnosed by NBS against year of birth. The red line indicates the universal rollout of NBS for cystic fibrosis across the UK. NBS, newborn bloodspot screening.

Since 2007 around 70% of all children born with CF in a given year had been diagnosed by NBS (figure 2).

### Association of diagnosis by NBS and health outcomes

We found significant main effects of NBS on weight-for-age SD-score, %FEV_1_, and time to cPA and a significant interaction effect between NBS and age on weight-for-age SD-score (table 2). Therefore, the best fitting model for the weight trajectories included a NBS main effect and interaction effect with age; for %FEV_1_ and cPA the best fitting model included an NBS main effect. We found no evidence that BMI SD-scores differed between individuals diagnosed by NBS and those diagnosed clinically.

**Table 2:** Parameter estimates (95% CIs) and likelihood ratio test (LRT) p values for the effect of diagnosis by NBS on weight-for-age SD-score trajectories, BM-for-age SD-score trajectories, %predicted FEV_1_ and cPA after adjustment for sex, genotype, year of birth, ethnicity, diagnosis by MI and pancreatic insufficiency. All numbers were rounded to two digits

	Weight	BMI	%FEV_1_	cPA
	Model with NBS main effect only			
NBS effect estimate	0.11 (0.03, 0.19)	0.02 (−0.05, 0.09)	1.56 (0.1, 3.02)	1.31 (1.07, 1.62)
LRT p value	<0.01	0.54	0.04	<0.01
	Model with NBS main effect and interaction with age			
NBS effect estimate	0.16 (0.07, 0.26)	0.05 (−0.05, 0.14)	0.87 (−0.88, 2.62)	NA
NBS:age interaction effect estimate	−0.02 (−0.03, −0.00)	−0.01 (−0.02, 0.01)	0.24 (−0.1, 0.58)	NA
LRT p value*	0.02	0.42	0.16	NA

*Compared with model with NBS main effect only.

BMI, body mass index; cPA, chronic *Pseudomonas aeruginosa*; FEV_1_, forced expiratory volume in one second; NBS, newborn bloodspot screening.

**Figure 3:**
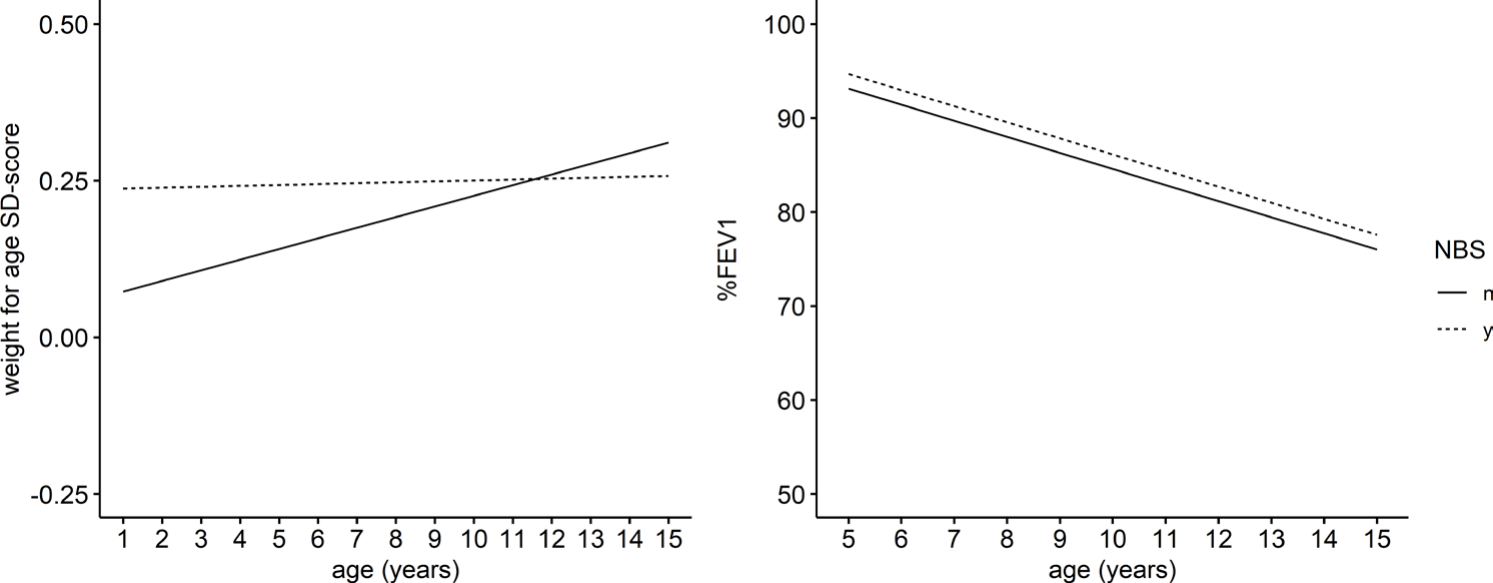
Expected weight for age SD-score and %FEV_1_ trajectories for individuals diagnosed by NBS (dotted line) and diagnosed clinically (solid line) with all other covariates set at reference levels (female, F508del homozygous, pancreatic sufficient, born in 2000, mean deprivation, white). FEV_1_, forced expiratory volume in one second; NBS, newborn bloodspot screening.

On average, individuals diagnosed by NBS had significantly higher weight-for-age SD-scores at age 1 (+0.16 SD-scores; 95% CI 0.07 to 0.26; equivalent to an increase of the population average weight from 53rd centile to the 59th centile for individuals with reference covariate values (female, F508del homozygous, white ethnicity, deprivation z-score of 0, pancreatic sufficient, born in 2000, no meconium ileus); see online supplementary material S5 for the interpretation of the results in other population subgroups). However, diagnosis by NBS was also associated with a marginally negative rate of weight SD-score change compared to the clinically diagnosed group (−0.02 95% CI −0.03 to −0.00) (table 2, figure 3). After 5 years, this corresponds to the average weight of individuals with reference covariate values diagnosed by NBS remaining in the 59th centile and the average weight in the clinically diagnosed group being in the 56th centile.

%FEV_1_ was higher in individuals diagnosed by NBS compared with those diagnosed clinically (+1.56 percentage points; 95% CI 0.1 to 3.02). There was no significant association between diagnosis by NBS and rate of change in lung function, therefore the estimated population average trajectories of those diagnosed by NBS and those diagnosed clinically ran in parallel over the age period we considered in this analysis (table 2, figure 3).

**Figure 4:**
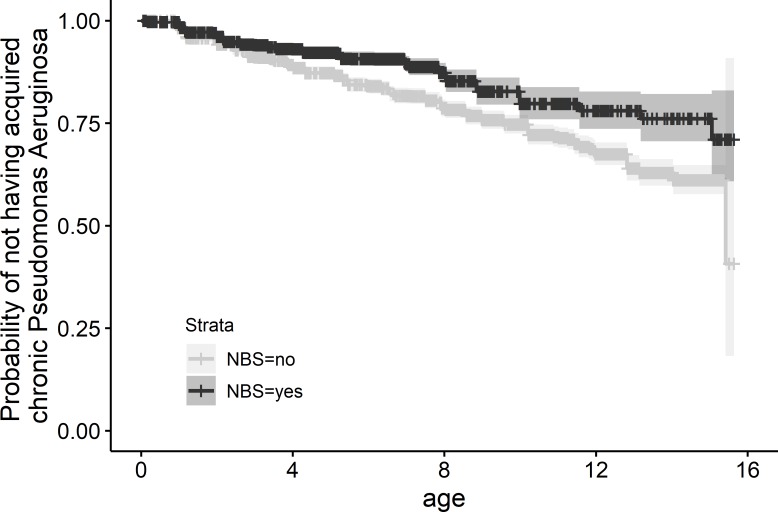
Non-parametric maximum likelihood estimator of the probability of not having acquired chronic *Pseudomonas aeruginosa* infection at any given age stratified by diagnosis by NBS. NBS, newborn bloodspot screening.

Diagnosis by NBS was associated with delayed cPA infection (table 2, figure 4). We found that in individuals diagnosed by NBS time to cPA infection was 1.3 times (95% CI 1.07 to 1.62) as long as in individuals diagnosed clinically. This corresponds to an estimated 16% chance of developing chronic PA infection before the age of 15 in individuals diagnosed by NBS compared with a 20% chance in those diagnosed clinically (with all other covariate values being set at their reference levels).

See section S6 in online supplementary material for parameters estimates for all the covariates included in the respective models.

### Estimation of inequalities in outcomes and the effect of NBS on these inequalities

The best fitting model for weight-for-age SD-scores included a deprivation main effect and an interaction with age; for %FEV_1_ the best fitting model only included a deprivation main effect. We did not find an association of deprivation with time to cPA and no main effect of deprivation on BMI though there may be differential change in BMI over time dependent on SECs (table 3).

**Table 3:** Parameter estimates (95% CIs) and likelihood ratio test (LRT) p values for the effect of deprivation on weight-for-age SD-score trajectories, BMI SD-score trajectories, %predicted FEV_1_ and cPA after adjustment for sex, genotype, year of birth, ethnicity, deprivation, diagnosis by MI and pancreatic insufficiency. The model for weight was further adjusted for a main effect of NBS and an interaction effect of NBS and age; the models for %FEV_1_ and cPA were adjusted for an NBS main effect. All numbers were rounded to two digits

	Weight	BMI	%FEV_1_	cPA
	Model with IMD main effect only			
IMD effect estimate	−0.05 (−0.08, −0.02)	0.01 (−0.02, 0.04)	−1.42 (−1.99, −0.85)	0.94 (0.88, 1.02)
LRT p value	<0.01	0.52	<0.01	0.12
	Model with IMD main effect and interaction with age			
IMD effect estimate	−0.08 (−0.11, −0.04)	−0.02 (−0.06, 0.01)	−1.52 (−2.2, −1.16)	NA
IMD: age interaction effect estimate	0.01 (0.00, 0.01)	0.01 (0.00, 0.01)	0.03 (−0.1, 0.16)	NA
LRT p value*	<0.01	<0.01	0.62	NA

*Compared with model with IMD main effect only.

BMI, body mass index; cPA, chronic *Pseudomonas aeruginosa*; FEV_1_, forced expiratory volume in one second; IMD, Index of Multiple Deprivation.

Individuals from more deprived backgrounds (ie, higher IMD SD-scores) had lower weight-for-age SD-scores at age 1 (−0.08 SD per SD of deprivation; 95% CI −0.11 to −0.04) and we found a marginally positive associations between weight-for-age SD-score rate of change and deprivation meaning that weight decreased more slowly or increased faster in children from more disadvantaged areas (table 3, figure S4 in online supplementary material S7). This corresponds to the population average weight in the least deprived 5% of individuals being in the 57th centile on the growth chart at age 1 while the average weight for the most deprived 5% at age 1 is in the 44th centile (when all covariate values are set at the reference level and the clinically diagnosed population is considered); after 5 years the average weight of the least deprived 5% is estimated to be in the 58th centile while it is estimated to be in the 53rd centile for the most deprived 5%.

Individuals from more deprived backgrounds also had lower %FEV_1_ from age 5 to the end of follow-up (−1.42 percentage points per SD of deprivation; 95% CI −1.99 to −0.85); there was no significant change in this effect over time (table 3, figure S4 in online supplementary material S7).

Weight and lung function were thus the only outcomes for which we found significant effects of both NBS and deprivation. Therefore, we assessed whether NBS has reduced inequalities in these outcomes. None of the interactions between NBS and IMD reached statistical significance at the 5% level; inequalities in weight at age 1 and lung function trajectories persisted in the population diagnosed by NBS (table 4). However, there may be a small differential increase in the rate of weight SD-score change in individuals diagnosed by NBS, whereby more disadvantaged individuals lost weight more slowly or gained weight more quickly (+0.01 SD in weight per year per IMD SD; 95% CI 0.01 to 0.02) (figure S5 in online supplementary material S7).

**Table 4:** Parameter estimates (95% CIs) and likelihood ratio test (LRT) p values for the interaction effect of diagnosis by NBS and deprivation on weight-for-age SD-core trajectories and %predicted FEV_1_ after adjustment for sex, genotype, year of birth, ethnicity, deprivation, diagnosis by NBS, diagnosis by MI and pancreatic insufficiency. Also, the estimated associations (95% CIs) of SECs with outcomes in the clinically diagnosed population and the population diagnosed by NBS based on the estimated interaction effects. All numbers were rounded to two digits

	Weight	%FEV_1_
	Main effects	
NBS: IMD interaction effect estimate	0.01 (−0.06, 0.08)	0.05 (−1.14, 1.25)
IMD effect in clinically diagnosed population	−0.08 (−0.13, −0.03)	−1.44 (−2.14, −0.74)
IMD effect in population diagnosed by NBS	−0.07 (−0.12, −0.03)	−1.39 (−2.36, −0.41)
	Interaction effects with age	
NBS:age: IMD interaction effect estimate	0.01 (−0.00, 0.02)	NA
IMD: age interaction effect in clinically diagnosed population	0.00 (−0.00, 0.01)	NA
IMD: age interaction effect in population diagnosed by NBS	0.01 (0.01, 0.02)	NA
LRT p value*	0.17	0.89

*Compared with model with no NBS: IMD interaction effects.

cPA, chronic *Pseudomonas aeruginosa*; FEV_1_, forced expiratory volume in one second; IMD, Index of Multiple Deprivation; NBS, newborn bloodspot screening.

See section S8 in online supplementary material for the parameter estimates for all covariates.

### Robustness tests

In the population born before 2007 the estimated main effect of diagnosis by NBS on weight-for-age SD-score and the interaction effect with age were smaller and no longer significant at the 5% level (0.05 SD; 95% CI −0.1 to 0.2 and 0.002 SD per year; 95% CI −0.01 to 0.02), though the differences may be due to the smaller study population. The estimated effects on BMI SD-scores, %FEV_1_ and on time to cPA infection were comparable to our main results. Population details and all parameter estimates are given in sections S4 and S9 in online supplementary material. Effects estimated after the exclusion of individuals diagnosed by meconium ileus were comparable to our main results for BMI, %FEV_1_ and time to cPA. The main effect of NBS on weight SD-scores was also in-line with the findings from our main analysis; the interaction effect of NBS on weight change was comparable to our main result but no longer reached statistical significance at the 5% level (−0.01 95% CI −0.03 to 0.001) (see online supplementary material section S10). Adjustment for propensity scores only and in combination with covariates gave estimates for the NBS effects, SECs effects and NBS-SECs interaction effects which were comparable to our main findings for BMI SD-scores, %FEV_1_, time to cPA and the main effect of NBS on weight SD-scores. The effect of NBS on weight SD-score change were again in-line with our main finings but no longer reached statistical significance at the 5% level (−0.01 95% CI −0.03 to 0.003 and −0.01 95% CI −0.03 to 0.002, respectively). See online supplementary material section S11 and S11b for details.

Estimates for the effect of NBS on time to cPA infection were comparable between the different time to event models (see online supplementary material section S12).

## Discussion

We carried out longitudinal analyses of the association of diagnosis by NBS with health outcomes in CF. Children diagnosed by NBS for CF in the UK in the new millennium have better lung function, delayed onset of cPA infection and increased weight at age 1 compared with those diagnosed clinically, but there is some evidence that higher weight for screened babies may diminish over time. We also assessed whether NBS reduced inequalities in outcomes, which have previously been shown for infants and children with CF. We did not find significant improvements in lung function and weight inequalities early in life. However, our results suggest that screening may lead to a reduction in inequalities in nutritional status over longer periods by reducing the decline or increasing the incline in weight outcomes to a larger extent in children from more deprived backgrounds compared with those of higher SECs.

### Strengths and limitations

Before evaluating the implications of our findings, we note several important strengths and limitations. A key strength is that our study evaluated the impact of NBS in the whole UK CF population born after the new millennium. This large unselected population allowed us to estimate even small effects of NBS in the early years that may lead to substantial improvements in CF in adulthood. Despite adjustment for a range of important confounders in our analysis, a potential limitation is that the universal roll-out of NBS in 2007 means that the non-NBS group in the more recent years may not be a valid comparator. In order to assess whether this could have biassed our results, we conducted a number of robustness tests, all of which corroborate our main conclusions. Repeating the analysis using propensity score matching, an alternative method for adjusting for confounding, showed the same results. Subgroup analysis only including individuals born prior to 2007 reduced our sample size and the length of follow-up significantly, which may be partly the reason why we could no longer demonstrate significant effects on weight outcomes. The estimated effect on lung function at age 5 and time to cPA were however comparable with those in our main analysis.

A further potential limitation is the short follow-up. Small effects on the rate of change of outcomes may not be ascertainable until later in adulthood given the high inter-individual variability. The skewed distribution of the data towards the early years is an additional limitation (see online supplementary figure S7 and supplementary material section S13). We analysed the data assuming linear trends in time and thus estimated a constant rate of change for each outcome over the study period and a constant effect of NBS on the rate of change. The estimates are heavily based on trends in the early years where the most data are available and may not be representative of trends later in childhood and adolescence should they change over this period. This may explain the slightly counter-intuitive results for weight trajectories in figure 3. Thus, we are more certain about the relationships in the early years, and the weight trajectories in figure 3 should be projected with caution due to lack of data at later time points.

There is a slight imbalance in the proportion of children with migration background between the group diagnosed by NBS and that diagnosed clinically. For children born abroad access to care and treatments may differ from the UK standards in the early years before their move to the UK. For almost all of these children the country of birth was entered as ‘other’, therefore it was impossible to draw strong conclusions about this. However, overall only a small proportion of the children included in the analysis was born in another country, therefore we do not believe this has affected our results substantially.

### Comparison with other studies

Our findings corroborate previous studies that have suggested an impact of NBS on outcomes in CF. A Cochrane review evaluated six trials, two of which were RCTs (UK Trial 1991,^21^ Wisconsin Trial 1998^22 23^), on NBS but only analysed data from one of the trials (Wisconsin Trial 1998).^6^ The review suggested that NBS leads to transient nutritional benefits in children with CF but that long-term effects on lung function are unclear. The review further found that screened participants were colonised with PA earlier than those diagnosed clinically. This is in contrast to our findings of delayed onset of chronic PA in those diagnosed by NBS, which may reflect more contemporary approaches to preventing cross infection. Similarly, a registry-based study in Canada in 2016 found lower incidence of PA and Staphylococcus aureus infection in individuals diagnosed by NBS compared with those diagnosed clinically.^24^ They also found that mean weight-for-age SD-score and height-for-age SD-score were higher in the population diagnosed by NBS compared with those diagnosed clinically with no difference in BMI-for-age, which is again in-line with our findings.

A number of other cross-sectional studies using the UK CF database, a pre-cursor of the UK CF registry, assessed the outcomes of individuals screened on the basis of regional programmes prior to universal coverage in the UK compared with those diagnosed symptomatically. The first of these studies suggested significantly greater median height and a reduction in morbidity in screened patients as compared with controls matched for age and genotype,^25^ and two subsequent studies suggested newborn screening led to less acute treatment requirements,^26^ and reduced healthcare costs.^27^ These findings were corroborated in another study by the same group that demonstrated improved height SD scores, but no difference in lung function, comparing screened to clinically diagnosed patients.^28^

### Clinical implications

Our findings show that diagnosis by NBS is associated with improved outcomes in early weight and lung function and delayed cPA infection. As discussed above we believe that the positive effect of NBS on weight outcomes may dissipate over time such that screened and unscreened children follow similar weight for age SD-score trajectories after the first few years of life. The positive effect of NBS on early lung function could be due to less obstruction or simply due to better lung growth in the early years.^29 30^ The effect appeared to be sustained throughout childhood indicating that it may have a lasting impact on long-term lung health and quality of life. Similarly, the estimated delay in onset of chronic Pseudomonas may have a positive effect on CF disease progression over time, since cPA acquisition is associated with a significantly increased rate of decline of lung function subsequently.^31^ However, we found no difference in the rate of decline between individuals diagnosed by NBS and those diagnosed clinically during the period we studied.

We have previously documented significant inequalities in health outcomes for people with CF in the UK^10^; similar findings were also noted in the USA.^32 33^ These early inequalities were again evident for the children in this study. The universal implementation of NBS in the UK ensures that all children are diagnosed within the first weeks of life. This removes any differential time to diagnosis and differential disease progression prior to diagnosis by socioeconomic status, whereby more disadvantaged children may become sicker prior to diagnosis in unscreened populations. The results of this study did not show any evidence of a narrowing in inequalities in lung function after NBS was implemented. Similarly, it did not show significant reductions in the inequalities in weight at age 1. However, we found a potentially differential effect on weight rate of change in the screened population. This suggests that we may see a narrowing of the inequalities gap in nutritional status over time. These results are complex and may reflect differential access to certain elements of care for families from less well-resourced backgrounds. It is possible that less disadvantaged families are able to better capitalise on the potential of an early diagnosis through better access to healthcare opportunities. There is evidence that healthcare practitioners interact more fully and spend more time with families from higher SECs^34^ and this possibility should also be considered when reflecting on this lack of narrowing of the health inequality gap. More research is needed to understand pathways to inequalities for children with CF. In the meantime, action to address poverty and improve social conditions for disadvantaged children growing up with CF is required to address health inequalities, even within the era of universal NBS.

In terms of generalisability it is worth noting that the data analysed here were largely from an era prior to the introduction of disease modifying drugs. It is conceivable that early diagnosis through NBS will have an even larger positive impact if access to CFTR modulators is immediately available and damage to the lungs and digestive system can be prevented. However, it will be essential to ensure equal access to these new treatments for all children independent of socioeconomic background to prevent a widening of health inequalities.

## Conclusion

In the UK, diagnosis of CF by NBS is associated with improvements in early measures of nutritional status and lung function that may translate into important health gains over the life-course. Our study does not suggest a dramatic reduction in health inequalities after expansion of NBS across the whole of the UK. Further research after longer follow-up may demonstrate an improvement in inequalities over time, but these results suggest that a deeper understanding of the drivers of health inequalities in CF is urgently required such that the underlying factors can be addressed.
