# Supplementary material for: Impact of newborn screening on outcomes and social inequalities in cystic fibrosis: a UK CF registry-based study
Source: Thorax. 2019 Nov 26;75(2):123–31. doi: 10.1136/thoraxjnl-2019-213179 (PMC7029232; doi:10.1136/thoraxjnl-2019-213179)
Supplement: Supplementary data [file thoraxjnl-2019-213179supp001.pdf]

Impact of newborn screening on outcomes and social inequalities in cystic fibrosis: A UK Registry based study

Supplementary Material

S1 Plots of the data

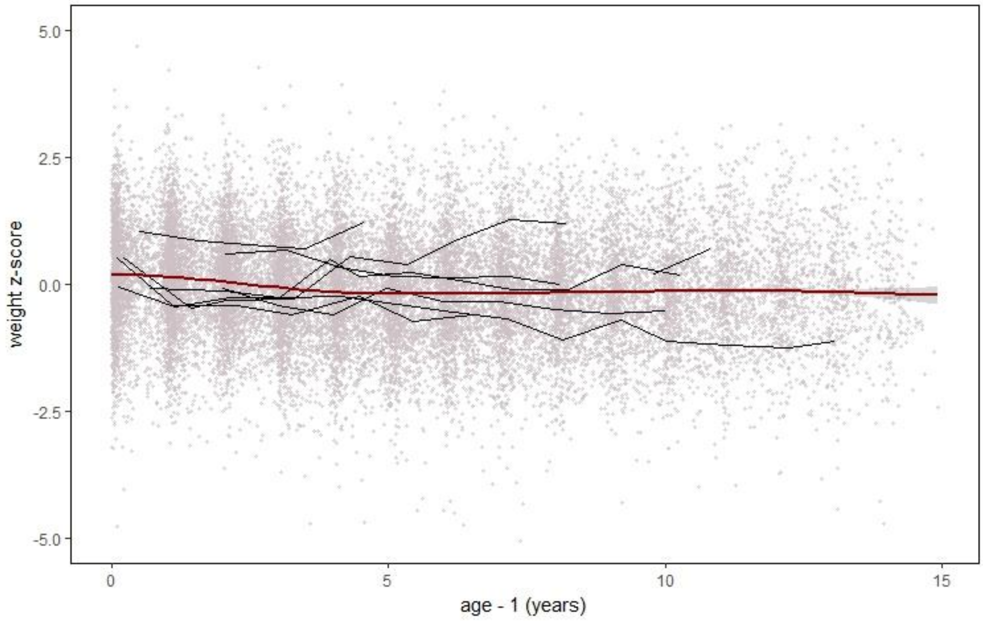

Figure S1: Plot of the longitudinal weight for age z-score data used in the analysis. Each grey dot represents one measurement. The black lines are trajectories for 10 randomly selected individuals. The red line is a smoother through the data.

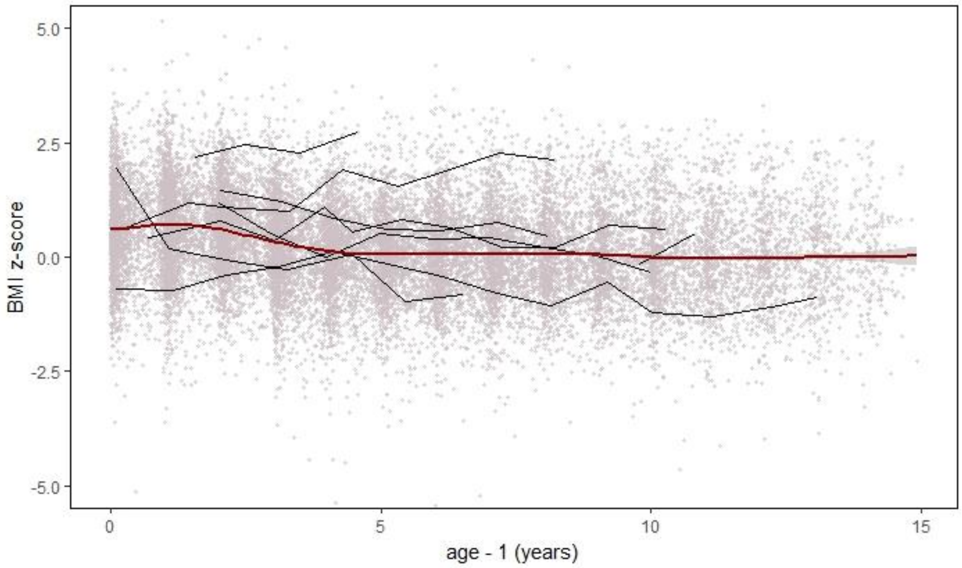

Figure S2: Plot of the longitudinal BMI z-score data used in the analysis. Each grey dot represents one measurement. The black lines are trajectories for 10 randomly selected individuals. The red line is a smoother through the data.

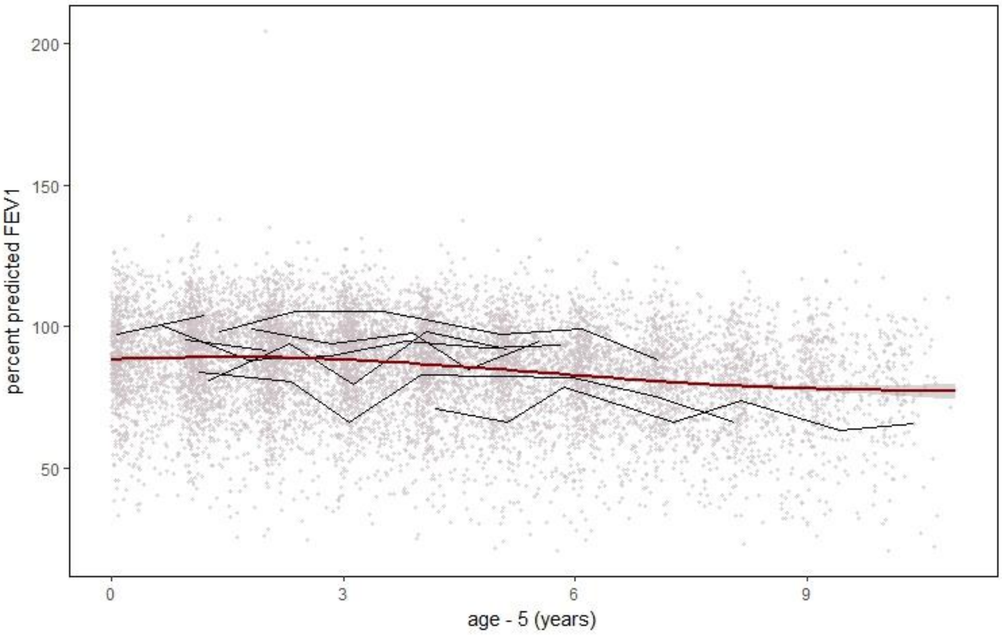

Figure S3: Plot of the longitudinal percent predicted FEV1 data used in the analysis. Each grey dot represents one measurement. The black lines are trajectories for 10 randomly selected individuals. The red line is a smoother through the data.

S2 Data cleaning

Diagnosis by NBS and meconium ileus

In cases where both, a diagnosis by NBS and a diagnosis by meconium ileus (MI) were indicated, we treated the diagnosis by MI as the actual method of diagnosis as this would happen before the results from the NBS were received.

Deprivation scores

We used small area deprivation measures as a proxy for childhood socio-economic conditions at birth. The deprivation score was generated from the family home address, based on the index of multiple deprivation (IMD). The first postcode recorded on the Registry was used and matched with Lower Super Output Areas (LSOA) in England and Wales (2011 Census data), Data Zones in Scotland (2001) and the Super Output Areas from Northern Ireland (2001). We used the English IMD 2015 score, the Welsh IMD 2014 score, the Scottish SIMD 2012 score and the Northern Irish NIMDM score 2010. In order to obtain a comparable measure of deprivation across the devolved nations, we calculated country-specific IMD z-scores, which we used in our analysis.

S3 Model equations and estimation of the effects of IMD in population diagnosed by NBS

Let y_{ij} denote the jth observed value for weight for age z-scores, BMI z-scores or % predicted FEV_1 in individual i. We model the longitudinal trajectories by

$$y_{ij} = x_i'\beta_1 + x_i't_{ij}\beta_2 + V_i + W_i t_{ij} + Z_{ij}$$

where x_i is the vector of exposures and baseline covariates for individual i (diagnosis by NBS, IMD z-score, year of birth, sex, genotype, pancreatic insufficiency, ethnicity, diagnosis by meconium ileus; in the model with the interaction effect between NBS and IMD in addition: NBS*IMD z-score),

t_{ij} is time of observation j in individual i measured as time since age 1 or 5

V_i and W_i are a random intercept and slope with (V_i,W_i)~BVN((0,0) , Σ)

Z_{ij} is measurement error with Z_{ij}~$N(0,\sigma_Z^2)$

Let T_i be the time at which individual i acquires chronic PA infection. We model the distribution of T using the Weibull distribution with the following survivor function

$$P(T > t) = S(t) = exp(-(t/\lambda)^p)$$

where λ = exp($x_i'\beta_3$) with x_i as above. The parameter estimates given in the main paper are the exponentiated coefficients; in the supplementary material we give $\hat{\beta}_3$. 1/p is the estimated scale parameter.

Let $\hat{\beta}_{IMD}$ and $\hat{\beta}_{NBS*IMD}$ be the estimated effect of IMD and the interaction effect between IMD and NBS, respectively. The effect of IMD in the population diagnosed by NBS is given by $\hat{\beta}_{IMD}$+$\hat{\beta}_{NBS*IMD}$, with Var($\hat{\beta}_{IMD}$+$\hat{\beta}_{NBS*IMD}$)=Var($\hat{\beta}_{IMD}$)+Var($\hat{\beta}_{NBS*IMD}$)+2*Cov($\hat{\beta}_{IMD}$,$\hat{\beta}_{NBS*IMD}$).

S4 Baseline demographics of the study population split by year of birth pre and post 2007

Table S1: Baseline demographics of the study population for the weight for age SD-score analysis split into those born before 2007 and after 2007

	Born before 2007		Born after 2007	
	Clin. Diag.	Diag. by NBS	Clin. Diag.	Diag. by NBS
n	1192	289	607	1179
male (%)	591 (49.6)	151 (52.2)	313 (51.6)	606 (51.4)
Non-white ethnicity (%)	68 (5.7)	11 (3.8)	48 (7.9)	42 (3.6)
diagnosis by meconium ileus (%)	293 (24.6)	0 (0.0)	303 (49.9)	0 (0.0)
F508 class (%)				
Heterozygous	388 (32.6)	115 (39.8)	220 (36.2)	480 (40.7)
Homozygous	686 (57.6)	151 (52.2)	319 (52.6)	605 (51.3)
Other	118 (9.9)	23 (8.0)	68 (11.2)	94 (8.0)
IMD z-score (median [IQR])	-0.29 [-0.79, 0.48]	-0.32 [-0.79, 0.59]	-0.29 [-0.73, 0.59]	-0.29 [-0.79, 0.70]
pancreatic insufficient (%)	1072 (89.9)	256 (88.6)	520 (85.7)	971 (82.4)

Table S2: Baseline demographics of the study population for the BMI SD-score analysis split into those born before 2007 and after 2007

	Born before 2007		Born after 2007	
	Clin. Diag.	Diag. by NBS	Clin. Diag.	Diag. by NBS
n	1192	289	600	1171
male (%)	591 (49.6)	151 (52.2)	309 (51.5)	603 (51.5)
Non-white ethnicity (%)	68 (5.7)	11 (3.8)	48 (8.0)	41 (3.5)
diagnosis by meconium ileus (%)	293 (24.6)	0 (0.0)	300 (50.0)	0 (0.0)
F508 class (%)				
Heterozygous	388 (32.6)	115 (39.8)	215 (35.8)	474 (40.5)
Homozygous	686 (57.6)	151 (52.2)	318 (53.0)	604 (51.6)
Other	118 (9.9)	23 (8.0)	67 (11.2)	93 (7.9)
IMD z-score (median [IQR])	-0.29 [-0.79, 0.48]	-0.32 [-0.79, 0.59]	-0.29 [-0.73, 0.57]	-0.29 [-0.79, 0.70]
pancreatic insufficient (%)	1072 (89.9)	256 (88.6)	517 (86.2)	967 (82.6)

Table S3: Baseline demographics of the study population for the %FEV1 analysis split into those born before 2007 and after 2007

	Born before 2007		Born after 2007	
	Clin. Diag.	Diag. by NBS	Clin. Diag.	Diag. by NBS
n	1174	287	270	485
male (%)	580 (49.4)	151 (52.6)	132 (48.9)	250 (51.5)
Non-white ethnicity (%)	64 (5.5)	11 (3.8)	23 (8.5)	21 (4.3)
diagnosis by meconium ileus (%)	290 (24.7)	0 (0.0)	135 (50.0)	0 (0.0)
F508 class (%)				
Heterozygous	383 (32.6)	114 (39.7)	95 (35.2)	191 (39.4)
Homozygous	678 (57.8)	150 (52.3)	143 (53.0)	248 (51.1)
Other	113 (9.6)	23 (8.0)	32 (11.9)	46 (9.5)
IMD z-score (median [IQR])	-0.29 [-0.79, 0.53]	-0.33 [-0.79, 0.58]	-0.22 [-0.73, 0.71]	-0.29 [-0.79, 0.47]
pancreatic insufficient (%)	1058 (90.1)	255 (88.9)	235 (87.0)	404 (83.3)

Table S4: Baseline demographics of the study population for the time to chronic P. aerugonisa analysis split into those born before 2007 and after 2007

	Born before 2007		Born after 2007	
	Clin. Diag.	Diag. by NBS	Clin. Diag.	Diag. by NBS
n	1193	289	634	1237
male (%)	591 (49.5)	151 (52.2)	329 (51.9)	636 (51.4)
Non-white ethnicity (%)	68 (5.7)	11 (3.8)	48 (7.6)	46 (3.7)
diagnosis by meconium ileus (%)	293 (24.6)	0 (0.0)	318 (50.2)	0 (0.0)
F508 class (%)				
Heterozygous	389 (32.6)	115 (39.8)	236 (37.2)	500 (40.4)
Homozygous	686 (57.5)	151 (52.2)	330 (52.1)	638 (51.6)
Other	118 (9.9)	23 (8.0)	68 (10.7)	99 (8.0)
IMD z-score (median [IQR])	-0.29 [-0.79, 0.47]	-0.32 [-0.79, 0.59]	-0.29 [-0.73, 0.59]	-0.25 [-0.79, 0.72]
pancreatic insufficient (%)	1072 (89.9)	256 (88.6)	542 (85.5)	1020 (82.5)

S5 Conversion of the results for the weight outcome from sd-scores into centiles

Change in population average weight at age one in the group diagnosed by NBS compared to the clinically diagnosed group in centiles for selected population subgroups:

Males, F508del homozygous, white ethnicity, deprivation z-score of 0, pancreatic sufficient, born in 2000, no meconium ileus

NBS: 62nd centile; clinically diagnosed: 56th centile

Females, F508del heterozygous, white ethnicity, deprivation z-score of 0, pancreatic sufficient, born in 2000, no meconium ileus

NBS: 55th centile; clinically diagnosed: 49th centile

Males, F508del heterozygous, white ethnicity, deprivation z-score of 0, pancreatic sufficient, born in 2000, no meconium ileus

NBS: 58th centile; clinically diagnosed: 42nd centile

S6 Parameter estimates

In a model without any adjustment for covariates the estimated population average trajectories for weight-for-age and BMI SD-scores had an intercept at age one of 0.07 (95%CI 0.03 to 0.11) and 0.62 (95%CI 0.58 to 0.65), respectively, and an annual decline of -0.03 (95% CI -0.04 to -0.03) and -0.08 (95%CI -0.08 to -0.07) standard deviations, respectively. %FEV_1 at age five was estimated to be 89.87 (95%CI 89.16 to 90.57) with an annual decline of -0.92 (95%CI -1.05 to -0.79) (Table S1).

Table S4: Parameter estimates (95% confidence intervals) for the population average intercept and slope of the weight for age SD-core, BMI SD-score and %predicted FEV1 trajectories for the whole population without covariate adjustment.

	Weight	BMI	FEV
Intercept	0.07 (0.03, 0.11)	0.62 (0.58, 0.65)	89.87 (89.16, 90.57)
annual rate of decline	-0.03 (-0.04, -0.03)	-0.08 (-0.08, -0.07)	-0.92 (-1.05, -0.79)

The following tables give the estimated parameters for the final models described in the main paper.

Table S5: Parameter estimates, standard errors and p-values for the final model for weight for age SD-scores. All numbers were rounded to two significant digits.

	Value	Std.Error	p.value
	Main effects		
Intercept	0.073	0.079	0.35
Diagnosis by NBS =y	0.16	0.047	<0.01
Sex=male	0.075	0.038	0.047
F508 class=Heterozygous	-0.11	0.043	<0.01
F508 class=Other	-0.2	0.076	<0.01
Ethnicity=other	-0.29	0.094	<0.01
IMD[1] z-score	-0.078	0.018	<0.01
PI[2]	-0.18	0.063	<0.01
Year of birth	0.018	0.0051	<0.01
Diagnosis by MI[3]=yes	-0.013	0.055	0.81
Age	0.017	0.011	0.12
	Interaction effects with age		
Age:diagnosis by NBS=yes	-0.016	0.0066	0.019
Age: sex=male	0.011	0.0052	0.031
Age:F508 class=Heterozygous	0.012	0.006	0.049
Age:F508 class=Other	0.034	0.01	<0.01
Age:ethnicity=other	0.012	0.013	0.36
Age:IMD[1] z-score	0.0074	0.0025	<0.01
Age:PI[2]	-0.029	0.0091	<0.01
Age:year of birth	-0.0056	0.00086	<0.01
Age:diagnosis by MI[3] =yes	-0.013	0.0073	0.077

[1]Index of multiple deprivation
[2]Pancreatic insufficiency
[3]Meconium ileus

Table S6: Variance estimates for the random effects and correlation between the effect on the intercept and the slope in the models for weight for age SD-scores.

	Variance	StdDev	Corr
Intercept	1.00	1.00	
Age	0.01	0.11	-0.46
Residual	0.14	0.38	

Table S7: Parameter estimates, standard errors and p-values for the final model for BMI SD-scores. All numbers were rounded to two significant digits.

	Value	Std.Error	P value
	Main effects		
Intercept	0.44	0.078	<0.01
Sex=male	0.15	0.037	<0.01
F508 class=Heterozygous	-0.11	0.043	0.013
F508 class=Other	-0.16	0.076	0.031
Ethnicity=other	-0.44	0.093	<0.01
IMD[1] z-score	-0.021	0.018	0.23
PI[2]	0.065	0.063	0.3
Year of birth	0.024	0.0045	<0.01
Diagnosis by MI[3]=yes	-0.18	0.048	<0.01
Age	-0.012	0.012	0.3
	Interaction effects with age		
Age: sex=male	0.0053	0.0056	0.35
Age:F508 class=Heterozygous	0.01	0.0065	0.11
Age:F508 class=Other	0.036	0.011	<0.01
Age:ethnicity=other	0.012	0.014	0.38
Age:IMD[1] z-score	0.0083	0.0028	<0.01
Age:PI[2]	-0.042	0.01	<0.01
Age:year of birth	-0.0075	0.00084	<0.01
Age:diagnosis by MI[3] =yes	0.0028	0.0072	0.69

[1]Index of multiple deprivation
[2]Pancreatic insufficiency
[3]Meconium ileus

Table S8: Variance estimates for the random effects and correlation between the effect on the intercept and the slope in the models for BMI SD-scores.

	Variance	StdDev	Corr
Intercept	0.87	0.93	
Age	0.01	0.11	-0.52
Residual	0.27	0.52	

Table S9: Parameter estimates, standard errors and p-values for the final model for %FEV1. All numbers were rounded to two significant digits.

	Value	Std.Error	p.value
	Main effects		
Intercept	93	1.5	0
Diagnosis by NBS =y	1.6	0.74	0.036
Sex=male	0.38	0.71	0.59
F508 class=Heterozygous	-0.81	0.82	0.32
F508 class=Other	-0.83	1.4	0.56
Ethnicity=other	-3.2	1.7	0.07
IMD[1] z-score	-1.4	0.29	<0.01
PI[2]	-5.1	1.3	<0.01
Year of birth	0.012	0.13	0.93
Diagnosis by MI[3]=yes	0.67	0.96	0.48
Age	-1.7	0.28	<0.01
	Interaction effects with age		
Age: sex=male	0.26	0.13	0.046
Age:F508 class=Heterozygous	0.45	0.15	<0.01
Age:F508 class=Other	0.32	0.27	0.23
Age:ethnicity=other	0.13	0.32	0.68
Age:PI[2]	-0.0389	0.25	0.87
Age:year of birth	0.23	0.03	<0.01
Age:diagnosis by MI[3] =yes	-0.032	0.16	0.84

[1]Index of multiple deprivation
[2]Pancreatic insufficiency
[3]Meconium ileus

Table S10: Variance estimates for the random effects and correlation between the effect on the intercept and the slope in the models for %FEV1.

	Variance	StdDev	Corr
Intercept	193.76	13.92	
Age	2.97	1.72	-0.4
Residual	76.71	8.76	

Table S11: Parameter estimates, standard errors and p-values for the model for time to chronic PA infection. All numbers were rounded to two significant digits.

	Value	Std.Error	p
Intercept	4.2	0.23	<0.01
Diagnosis by NBS=yes	0.28	0.1	<0.01
Sex=male	0.1	0.078	0.2
PI[1]	-1.1	0.21	<0.01
F508 class=Heterozygous	0.036	0.088	0.68
F508 class=other	0.21	0.16	0.2
Ethnicity=other	-0.57	0.16	<0.01
Year of birth	0.062	0.015	<0.01
Diagnosis by MI[2]=yes	0.062	0.1	0.54
Log(Scale)	-0.046	0.042	0.28

[1]Pancreatic insufficiency
[2]Meconium ileus

S7 Expected weight for age SD-score and %FEV1 trajectories depending of socio-economic conditions

Figures S4 and S5 show the expected weight for age SD-score and %FEV1 trajectories for the least and most deprived 5% of the study population without and with taking into account potential differential effects of the screening program estimated in the weight analysis. IT is important to note here again that the estimates are heavily based on trends in the early years where the most data are available and may not be representative of trends later in childhood and adolescence should they change over this period.

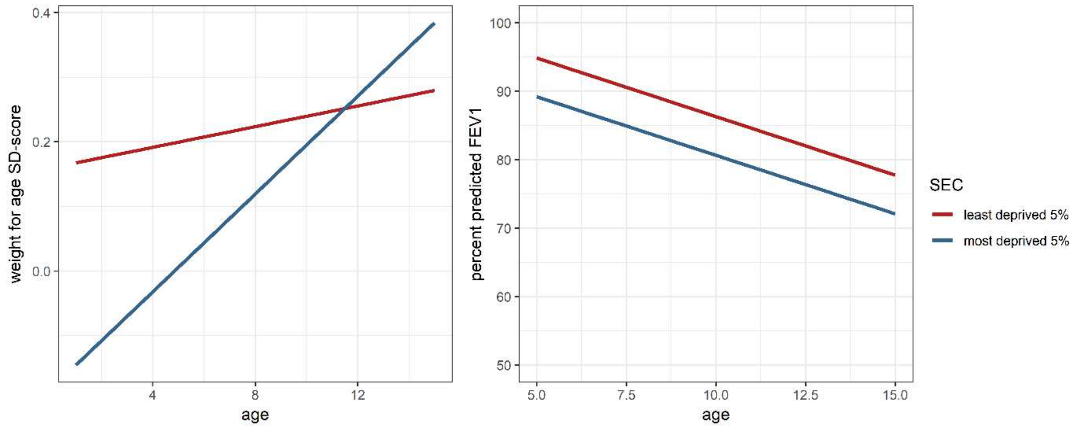

Figure S4: Expected weight for age SD-score and %FEV$_1$ trajectories for individuals from the least and most deprived 5% of the study population that were diagnosed clinically based on the model which does not include interaction effects between NBS and IMD (all other covariates set at their reference levels: female, F508del homozygous, pancreatic sufficient, born in 2000, white).

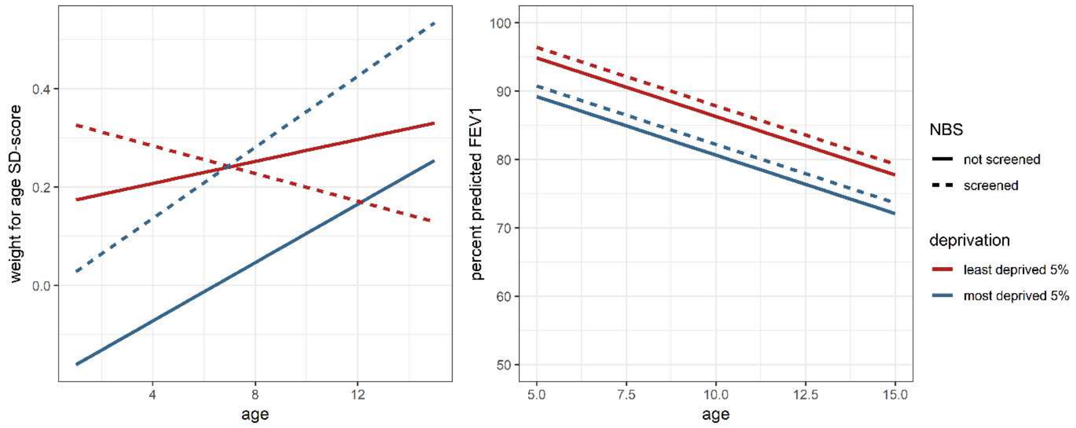

Figure S5: Expected weight for age SD-score and %FEV1 trajectories for individuals from the least and most deprived 5% of the study population that were diagnosed clinically or through screening based on the model that included interaction terms between IMD SD-scores and diagnosis by NBS (all other covariates set at their reference levels: female, F508del homozygous).

S8 Parameter estimates from models that include NBS-deprivation interaction terms

Table S12: Parameter estimates, standard errors and p-values for the models for weight for age SD-scores. All numbers were rounded to two significant digits.

	Value	Std.Error	p-value
	Main effects		
Intercept	0.073	0.079	0.35
Diagnosis by NBS=yes	0.16	0.047	<0.01
Sex=male	0.075	0.038	0.047
F508 class=Heterozygous	-0.11	0.043	<0.01
F508 class=Other	-0.2	0.076	<0.01
Ethnicity=other	-0.29	0.094	<0.01
IMD[1] z-score	-0.083	0.025	<0.01
PI[2]	-0.18	0.063	<0.01
Year of birth	0.018	0.0051	<0.01
Diagnosis by MI[3]=yes	-0.014	0.055	0.8
NBS: IMD[1] z-score	0.0093	0.036	0.79
Age	0.017	0.011	0.13
	Interaction effects with age		
Age:diagnosis by NBS=yes	-0.016	0.0066	0.018
Age: sex=male	0.011	0.0052	0.031
Age: F508 class=Heterozygous	0.012	0.006	0.049
Age: F508 class=Other	0.034	0.01	<0.01
Age:ethnicity=other	0.012	0.013	0.35
Age:IMD[1] z-score	0.0046	0.0033	0.16
Age: PI[2]	-0.028	0.0091	<0.01
Age:year of birth	-0.0055	0.00086	<0.01
Age:diagnosis by MI[3]	-0.013	0.0073	0.07
Age:NBS:IMD[1] z-score	0.0079	0.0052	0.13

[1]Index of multiple deprivation
[2]Pancreatic insufficiency
[3]Meconium ileus

Table S13: Variance estimates for the random effects and correlation between the effect on the intercept and the slope in the models for weight for age SD-scores.

	Variance	StdDev	Corr
Intercept	1.00	1.00	
Age	0.01	0.11	-0.46
Residual	0.14	0.38	

Table S14: Parameter estimates, standard errors and p-values for the models for %FEV1. All numbers were rounded to two significant digits.

	Value	Std.Error	p-value
	Main effects		
Intercept	93	1.5	0
Diagnosis by NBS=yes	1.6	0.74	0.037
Sex=male	0.38	0.71	0.59
F508 class=Heterozygous	-0.81	0.82	0.32
F508 class=Other	-0.83	1.4	0.56
Ethnicity=other	-3.2	1.7	0.07
IMD[1] z-score	-1.4	0.36	<0.01
PI[2]	-5.1	1.3	<0.01
Year of birth	0.012	0.13	0.93
Diagnosis by MI[3]=yes	0.67	0.96	0.48
NBS: IMD[1] z-score	0.052	0.61	0.93
Age	-1.7	0.28	<0.01
	Interaction effects with age		
Age: sex=male	0.26	0.13	0.046
Age: F508 class=Heterozygous	0.45	0.15	<0.01
Age: F508 class=Other	0.32	0.27	0.23
Age:ethnicity=other	0.13	0.32	0.68
Age: PI[2]	-0.039	0.25	0.87
Age:year of birth	0.23	0.03	<0.01
Age:diagnosis by MI[3]	-0.032	0.16	0.84
[1]Index of multiple deprivation<br>[2]Pancreatic insufficiency<br>[3]Meconium ileus			

Table S15: Variance estimates for the random effects and correlation between the effect on the intercept and the slope in the models for %FEV1.

	Variance	StdDev	Corr
Intercept	193.75	13.92	
Age	2.97	1.72	-0.4
Residual	76.71	8.76	

S9 Effect estimates from Robustness test in which we only included individuals born before 2007

In the Robustness test we included only individuals born between 2000 and 2007. For the analysis of the weight for age z-scores and the BMI z-score this was the case for 1481 individuals out of which 289 individuals were diagnosed by NBS. For the analysis of %FEV1 1461 individuals were born before 2007 out of which 287 individuals were diagnosed by NBS. For the analysis of time to chronic PA, 1482 individuals were born before 2007 (289 diagnosed by NBS) out of which 460 had events.

Table S16: Parameter estimates, 95% confidence intervals and likelihood ratio test p-values for the effect of NBS on outcomes in individuals born before 2007 when NBS for CF was not universally available. All estimates rounded to two digits.

	Weight	BMI	%FEV1	cPA
	Model with NBS main effect only			
NBS effect estimate	0.06(-0.06, 0.18)	-0.06 (-0.18, 0.05)	2.08 (0.23, 3.94)	1.31 (1.02, 1.7)
LRT p-value[1]	0.34	0.28	0.03	0.03
	Model with NBS main effect and interaction with age			
NBS effect estimate	0.05 (-0.1, 0.2)	-0.14 (-0.29, 0.01)	1.75 (-0.53, 4.03)	NA
NBS:age interaction effect estimate	-0.00 (-0.01, 0.02)	0.01 (-0.00, 0.03)	0.09 (-0.27, 0.45)	NA
LRT p-value[2]	0.83	0.1	0.62	NA

[1] Likelihood ratio test p-value from comparison with model with no adjustment for NBS
[2] Likelihood ratio test p-value from comparison with model with NBS main effect only

S10 Effect estimates from robustness test in which we excluded individuals diagnosed by meconium ileus

In this robustness test we excluded individuals who were diagnosed by meconium ileus as by construction all of these individuals fell into the clinically diagnosed group. In the main analysis we already adjusted for meconium ileus, however in this robustness test we double checked that there was no bias introduced by this group. For the analysis of the weight for age z-scores and the BMI z-scores 2671 and 2659 individuals were included in the analysis, respectively, out of which 1468 and 1460 individuals were diagnosed by NBS. For the analysis of %FEV1 we included 1468 out of which 772 individuals were diagnosed by NBS. For the analysis of time to chronic PA, 2742 individuals were not diagnosed by meconium ileus (1216 diagnosed by NBS) out of which 475 had events.

Table S17: Parameter estimates, 95% confidence intervals and likelihood ratio test p-values for the effect of NBS on outcomes under exclusion of individuals diagnosed by meconium ileus. All estimates rounded to two digits.

	Weight	BMI	%FEV1	cPA
	Model with NBS main effect only			
NBS effect estimate	0.1 (0.02, 0.18)	0.02 (-0.06, 0.1)	1.7 (0.16, 3.19)	1.33 (1.08, 1.64)
LRTp-value[1]	0.02	0.63	0.03	<0.01
	Model with NBS main effect and interaction with age			
NBS effect estimate	0.14 (0.05, 0.24)	0.04 (-0.06, 0.13)	1.01 (-0.81, 2.83)	NA
NBS:age interaction effect estimate	-0.01 (-0.03, 0.00)	-0.01 (-0.02, 0.01)	0.22 (-0.11, 0.56)	NA
LRT p-value[2]	0.07	0.51	0.2	NA

[1] Likelihood ratio test p-value from comparison with model with no adjustment for NBS
[2] Likelihood ratio test p-value from comparison with model with NBS main effect only

S11 Parameter Estimates from robustness test in which we adjusted for propensity scores only

In order to check whether any imbalance in covariate values between the group diagnosed by NBS and that diagnosed clinically was appropriately adjusted for by covariate adjustment in the main analysis, we repeated the analysis adjusting for propensity scores; both on their own and together with covariate adjustment.

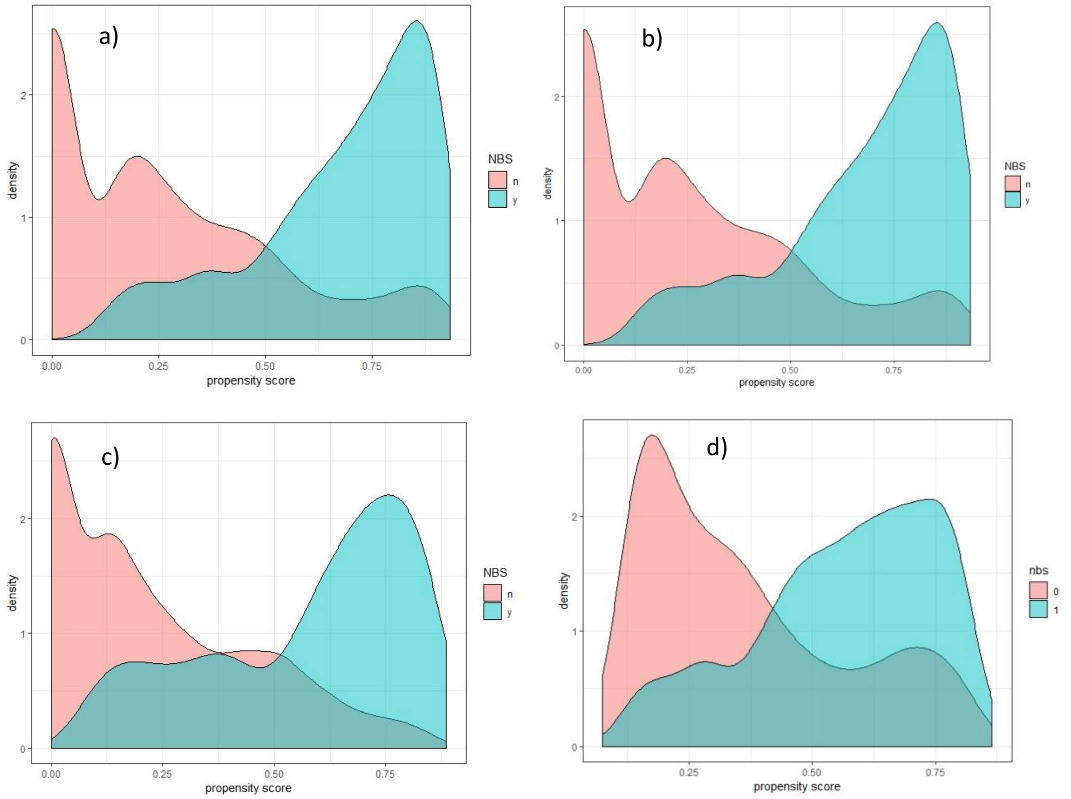

Figure S6: Distribution of probability of being diagnosed by NBS in the groups diagnosed by NBS and diagnosed clinically for the four outcomes: a) weight, b) bmi , c) %FEV1, d) cPA.

Table 18: Parameter estimates, 95% confidence intervals and likelihood ratio test p-values for the effect of NBS on outcomes after adjustment for propensity scores only.

	Weight	BMI	%FEV1	cPA
	Model with NBS main effect only			
NBS effect estimate	0.1 (0.01, 0.18)	0.01 (-0.07, 0.09)	1.6 (0.08, 3.15)	1.29 (1.05, 1.57)
LRT p-value[1]	0.02	0.8	0.04	<0.01
	Model with NBS main effect and interaction with age			
NBS effect estimate	0.14 (0.04, 0.23)	0.02 (-0.08, 0.11)	1.16 (-0.67, 3)	NA

NBS:age interaction effect estimate	-0.01 (-0.03, 0.00)	-0.01 (-0.02, 0.01)	0.15 (-0.2, 0.5)	NA
LRT p-value[2]	0.11	0.69	0.39	NA
[1] Likelihood ratio test p-value form comparison with model without adjustment for NBS<br>[2] Likelihood ratio test p-value from comparison with model with NBS main effect only				

Table S19: Parameter estimates, 95% confidence intervals and likelihood ratio test p-values for the effect of IMD on weight and %FEV1 after adjustment for propensity scores only.

	Weight	%FEV1
	Model with IMD main effect only	
IMD[1] effect estimate	-0.06 (-0.09, -0.03)	-1.52 (-2.1, -0.95)
LRT p-value[2]	<0.01	<0.01
	Model with IMD main effect and interaction with age	
IMD[1] effect estimate	-0.08 (-0.12, -0.05)	-1.58 (-2.27, -0.9)
IMD[1]:age interaction effect estimate	0.01 (0.00, 0.01)	0.02 (-0.1, 0.16)
LRT p-value[2]	<0.01	0.73
[1] Index of multiple deprivation<br>[2] Likelihood ratio test p-value from comparison with model without adjustment for IMD<br>[3] Likelihood ratio test p-value from comparison with model with IMD main effect only		

Table S20: Parameter estimates, 95% confidence intervals and likelihood ratio test p-values for the IMD:NBS interaction effects and the resulting estimated effects of IMD on weight and %FEV1 in the screened and clinically diagnosed population after adjustment for propensity scores only.

	Weight	%FEV1
	Main effects	
NBS:IMD[1] interaction effect estimate	0.01 (-0.06, 0.08)	0.14 (-1.07, 1.35)
IMD[1] effect in clinically diagnosed population	-0.09 (-0.14, -0.04)	-1.57 (-2.27, -0.86)
IMD[1] effect in population diagnosed by NBS	-0.08 (-0.13, -0.03)	-1.39 (-2.37, -0.42)
	Interaction effects with age	
NBS:age:IMD[1] interaction effect estimate	0.01 (-0.00, 0.02)	NA
IMD[1]:age interaction effect in clinically diagnosed population	0.01 (-0.00, 0.01)	NA
IMD[1]:age interaction effect in population diagnosed by NBS	0.01 (0.01, 0.02)	NA
LRT p-value[2]	0.17	0.82
[1] Index of multiple deprivation<br>[2]Likelihood ratio test p-value from comparison with model without NBS:IMD interaction effect		

S11b Effect estimates from robustness test in which we adjusted for propensity scores in addition to the covariates

Table S21: Parameter estimates, 95% confidence intervals and likelihood ratio test p-values for the effect of NBS on outcomes after adjustment for propensity scores and covariates.

	Weight	BMI	%FEV1	cPA
	Model with NBS main effect only			
NBS effect estimate	0.1 (0.02, 0.19)	0.02 (-0.06, 0.09)	1.7 (0.19, 3.22)	1.33 (1.1, 1.62)
LRT p-value[1]	0.02	0.69	0.03	<0.01
	Model with NBS main effect and interaction with age			
NBS effect estimate	0.14 (0.05, 0.24)	0.03 (-0.07, 0.13)	1.15 (-0.67, 3)	NA
NBS:age interaction effect estimate	-0.01 (-0.03, 0.00)	-0.01 (-0.02, 0.01)	0.18 (-0.15, 0.53)	NA
LRT p-value[2]	0.1	0.63	0.28	NA

[1] Likelihood ratio test p-value of comparison with model without adjustment for NBS
[2] Likelihood ratio test p-value of comparison with model with NBS main effect only

Table S22: Parameter estimates, 95% confidence intervals and likelihood ratio test p-values for the effect of IMD on weight and %FEV1 after adjustment for propensity scores and covariates.

	Weight	%FEV1
	Model with IMD main effect only	
IMD[1] effect estimate	-0.05 (-0.08, -0.02)	-1.43 (-2, -0.86)
LRT p-value[2]	<0.01	<0.01
	Model with IMD main effect and interaction with age	
IMD[1] effect estimate	-0.08 (-0.11, -0.04)	-1.53 (-2.22, -0.84)
IMD[1]:age interaction effect estimate	0.01 (0.00, 0.01)	0.04 (-0.09, 0.16)
LRT p-value[2]	<0.01	0.58

[1]Index of multiple deprivation
2 Likelihood ratio test p-value from comparison with model without adjustment for IMD
[2] Likelihood ratio test p-value from comparison with model with IMD main effect only

Table S23: Parameter estimates, 95% confidence intervals and likelihood ratio test p-values for the IMD:NBS interaction effects and the resulting estimated effects of IMD on weight and %FEV1 in the screened and clinically diagnosed population after adjustment for propensity scores and covariates.

	Weight	%FEV1
	Main effects	
NBS:IMD[1] interaction effect estimate	0.01 (-0.06, 0.08)	0.05 (-1.14, 1.25)
IMD[1] effect in clinically diagnosed population	-0.08 (-0.13, -0.03)	-1.44 (-2.14, -0.74)

IMD[1] effect in population diagnosed by NBS	-0.08 (-0.12, -0.03)	-1.39 (-2.36, -0.41)
	Interaction effects with age	
NBS:age:IMD[1] interaction effect estimate	0.01 (-0.00, 0.02)	NA
IMD[1]:age interaction effect in clinically diagnosed population	0.00 (-0.00, 0.01)	NA
IMD[1]:age interaction effect in population diagnosed by NBS	0.01 (0.01, 0.02)	NA
LRT p-value[2]	0.17	0.93

[1] index of multiple deprivation

[2] Likelihood ratio test p-value from comparison with model without NBS:IMD interaction effects

S12 Parameter Estimates from robustness test in which we use different parametric assumptions to model time to chronic pseudomonas aeruginosa

Table S24: Parameter estimates, standard errors and p-values for the Weibull, exponential and log-logistic model for time to chronic PA infection. All numbers were rounded to two significant digits.

	Exponential Model			Weibull model			Log-logistic model		
	Value	Std.Error	p	Value	Std.Error	p	Value	Std.Error	p
Intercept	4.3	0.23	3.8e-77	4.2	0.23	4e-76	3.9	0.22	3.2e-70
Diagnosis by NBS=yes	0.29	0.11	0.0078	0.28	0.1	0.0084	0.26	0.11	0.015
Sex=male	0.1	0.082	0.22	0.1	0.078	0.2	0.093	0.083	0.26
PI	-1.2	0.21	3.8e-08	-1.1	0.21	7.3e-08	-1.1	0.2	4.1e-08
F508 class=Heterozygous	0.038	0.092	0.68	0.036	0.088	0.68	0.017	0.093	0.86
F508 class=other	0.22	0.17	0.2	0.21	0.16	0.2	0.26	0.17	0.13
Ethnicity=other	-0.59	0.17	0.00037	-0.57	0.16	0.0043	-0.68	0.18	0.00018
Year of birth	0.069	0.013	2.1e-07	0.062	0.015	2.2e-05	0.063	0.015	1.6e-05
Diagnosis by MI=yes	0.065	0.11	0.54	0.062	0.1	0.54	0.049	0.11	0.66
Log(Scale)	NA	NA	NA	-0.046	0.042	0.28	-0.14	0.041	0.00068

The figures below show the empirical survivor function and hazard for chronic pseudomonas infection (black lines) and the fitted models.

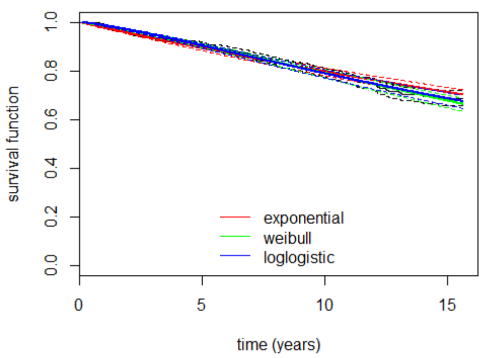

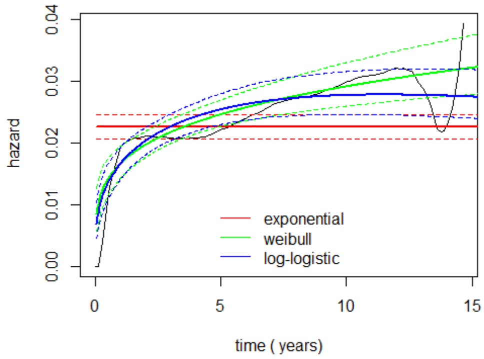

S13 Expected Trajectories and number of data points throughout the age range

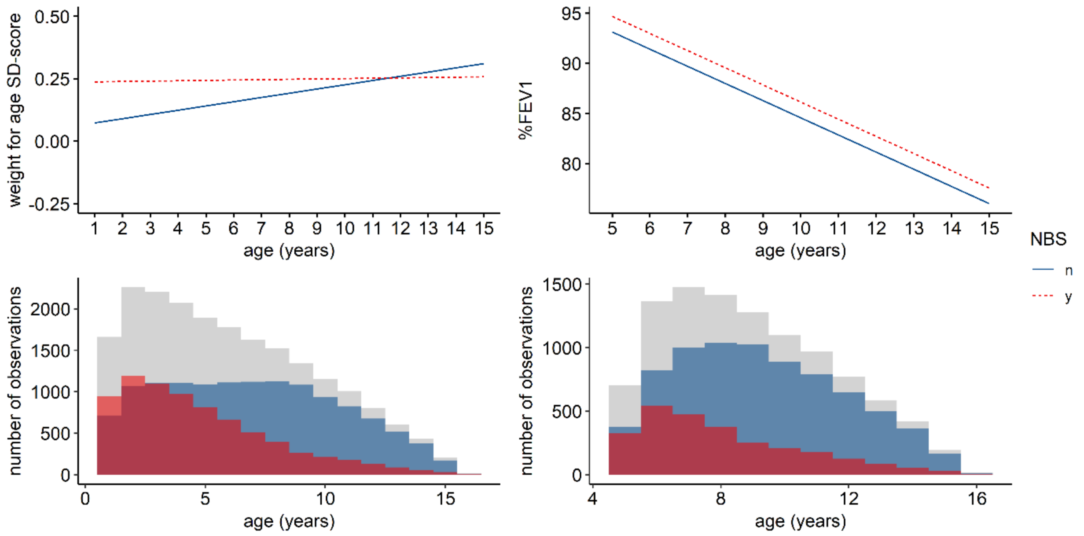

Figure S7: Top Panel: Expected weight for age SD-score trajectories for individuals diagnosed by NBS (red dotted line) and diagnosed clinically (blue solid line) with all other covariates set at reference levels (female, F508del heterozygous, pancreatic sufficient, born in 2000, mean deprivation, white). Bottom Panel: Distribution of the number of observations over the age range; grey: all observations, red: diagnosed by NBS, blue: diagnosed clinically.
